# Domestic Correspondence

**Published:** 1890-07

**Authors:** 


### Domestic Correspondence.

To the Editor:

*Refining Waste Amalgam.*—The question of the easiest and best method of refining amalgam scraps, either those which have been mixed up and not used, or old fillings, so that they can be again utilized, is of interest to every practising dentist.

It is well known that mercury boils at 357° C. and can be driven off from amalgam by a sufficiently high heat. The other metals present are not very easily volatile, and, if they can be prevented from oxidizing, should be left in a condition to again form an amalgam on the addition of mercury. I have made some experiments in this line, and find that this result can be obtained in the following manner.

The mercury may be allowed to escape, or the process can be conducted so as to preserve it. In the former case the operation should be carried on where there is a good draught to remove the vapors of mercury so as to escape the danger from breathing them. It is only necessary to heat the scraps in a crucible until the mercury is expelled, using a flux to prevent the oxidation of the alloy. A sand crucible may be employed and a coal-fire, or any heat by which it can be raised to redness. As a flux, borax glass—borax which has been fused to drive off the water of crystallization—answers very well. The amalgam should be placed in the hot crucible in small portions so that it may not be thrown out by the sudden conversion of the mercury into vapor. Enough borax should be used to form a ring when melted around the button of alloy, and the heat must be maintained until the mass has come to a quiet fusion and the globules of mercury at first seen on the walls of the crucible have disappeared. The metal can then be poured out and the ingot reduced to the necessary fineness by any of the common methods. As a convenient means of doing this I have been accustomed to pour the mass into an unglazed porcelain mortar which has been heated so that it can hardly be held in the hand and grinding quickly with a warm pestle. By a little practice the metal can be, by this means, reduced to a fine state. The coarser particles may be sifted out by a fine wire gauze and remelted. As some of the borax will probably remain mixed with the alloy, it is advisable to boil it a few minutes in water that this may be removed if present. After drying it is ready for use.

If the mercury is to be recovered, the scraps must be heated in a mercury retort or a crucible with a bent tube inserted through the cover. The mercury will distil over through this and can be condensed where there is no possibility of breathing the vapor. When as much as possible has been removed in this way the heating should be continued a short time with the crucible open in order to expel the last traces. The residue can then be treated as described above.

E. W. Rockwood,
*Demonstrator of Chemistry in the Dental Department State University of Iowa.*

Clinical use of amalgam proposed by the method above described has failed to show any material difference between it and the original alloy; if any, it is in its favor, as it seems to work more smoothly, and is rather more plastic. It has good edge-strength and good color and takes a good finish. By this method, which is simple, a considerable saving can be made by those who have been in the habit of throwing their amalgam scraps away or selling them to the dealers.

A. O. Hunt.

---

To the Editor:

*Mouth-Breathing.*—In discussing the various causes of irregularity of dental arches at the recent anniversary meeting of the First District Dental Society of New York, several of the speakers declared "mouth-breathing" to be one of the causes of these irregularities. It was argued that the force of pressure against the side teeth, produced by the muscles of the face, where the mouth is kept open so much of the time, tends to contract the arches by flattening them on either side and causing the incisors to protrude.

Many times have I observed that children with contracted dental arches were in the habit of breathing through their mouths, yet it never occurred to me that such deformities were produced in this manner. Whether such is a fact or not, I would hardly venture even an opinion; but this I can safely assert, that mouth-breathing is a habit as pernicious as it is unnatural. It tends to distort the entire expression of the countenance, giving a naturally fine-looking face a weary, ugly, and prematurely old appearance.

It is a wonder to me that so many persons have contracted and will permit themselves to continue so disgusting a habit. If they could but see themselves as others see them, with their lips rolling apart, their teeth (often uncleanly) and a large territory of gums exposed, together with pinched nostrils and the agonized expression of their faces, it seems to me that they would learn to put their noses to the use for which they were intended.

Mouth-breathing tends to keep the mucous membrane of the mouth and throat in a dry and irritated condition, and I often think favors the production of caries.

C. E. Francis, M.D.S.

New York.

---

To the Editor:

In much of the cranial development in the world it would seem that phrenologists might have considerable difficulty in finding anything to indicate the faculty expressed by "*why*" in the intellect of the owner. For in every walk of life we find mortals stumbling along, trying to find the pathway by the dim light of a few hastily-lighted and poorly-trimmed conclusions, the result being that mankind often finds a counterpart in the parrot, which can imitate a few things fairly well.

No man will excel in his occupation unless he knows the "why" of what he is doing. The stone-mason is no better than a machine, unless he knows the nature of the material he employs and why it brings certain results.

A "skilled" mechanic will not be skilled, unless he knows something of the "why" of what he is doing; that is, he must know the characteristics of the materials he uses and why certain results are obtained. Then, only, can he accomplish the highest and most perfect success.

Many students go through their school-life with "why" left out, and here it is, probably, that much of this defect originates,—in not developing more of the reasoning powers. Often it is that a child's lessons are so, because the book says so,—"why" it is thus, is to them an unfathomed sea.

In schools of medicine and of dentistry much of the same element is manifest. "How" usually occupies the front seats, while poor "why" is pushed to the outside. In professional life the same factor comes up again. A person is sick and is given a certain drug,—"why?" Oh, because a certain materia medica stated that it is one of the best drugs for that certain disease; or worse yet, as we have seen an M.D. do, copy his prescriptions from some text-book.

An alveolar abscess is presented for treatment, peroxide of hydrogen is used,—and "why?" Because it acts nicely in cleansing the cavity and was advised by Dr. ———. A patient comes with a tooth that is aching severely, and demands extraction, and it is done. Why did it ache? From irritation at some other part of the fifth nerve. The effect has been treated; the cause remains. An edentulous mouth needing artificial substitutes may present difficulties in the way of a satisfactory adaptation. A large, deep air-chamber is put in the upper case. This will usually hold the plate in position until the membranes and sometimes the hard parts fill the vacuum, when the teeth must be reset and a larger air-chamber made. If "why?" had been first employed, the model would have been scraped at the points offering the least resistance in the mouth, producing an even and continuous pressure from the plate, when, if any absorption occurs, it would be more nearly equal.

In dental conventions the first question in the discussion of a clinic or essay is too often, "Doctor, how do you do that?" It may be that some extreme (but true) illustrations have been here cited, and it will not be denied that "how?" is a little less prominent than in times past, but let us always give more attention to the cause, and in all our study and labors make foremost and most prominent, "why?"

M. G. Jenison, M.D.,D.D.S.

Minneapolis.

---

To the Editor:

At a meeting of the St. Louis Dental Society, held on Tuesday, May 20, 1890, the following resolutions on the death of Dr. Homer Judd were adopted:

Whereas, The members of St. Louis Dental Society have learned with great sorrow that death has removed from us our loved and honored associate, Dr. Homer Judd; and

Whereas, By reason of his great natural abilities, ripe scholarship, zeal, industry, and integrity, he was recognized by the dental profession as one of its most influential members; a man who devoted his life to the honor and advancement of his profession; and

WHEREAS, During a long professional life, his relations with this society have been such that it is our pleasure and duty to record our high appreciation of him; therefore

*Resolved*, That by the death of Dr. Homer Judd the dental profession has been deprived of one of its most able and useful members, one whose influence for good will live forever.

*Resolved*, That we extend to his family our sincere sympathy in their great bereavement.

*Resolved*, That a copy of these resolutions be sent to the family of the deceased and to the dental journals for publication.

W. H. EAMES,
HENRY FISHER,
A. J. PROSSER,
*Committee.*
MRS. EMMA EAMES CHASE,
*Cor. Secretary.*

---

TO THE EDITOR:

*The Dental Protective Association.*—The association is progressing nicely. We have a large membership and a large amount of testimony in regard to crown- and bridge-work antedating all patents owned by the D. P. C. Co. The company does not dare sue any member of the association nor proceed with old suits taken charge of by the association. We guarantee protection against any of their patent claims, and to take charge of all suits brought against members and pay expenses. The membership fee is only ten dollars, but will be increased soon.

J. N. CROUSE, *Chairman.*

